# A near-complete genome assembly of *Thalia dealbata* Fraser (Marantaceae)

**DOI:** 10.3389/fpls.2023.1183361

**Published:** 2023-06-13

**Authors:** Min Tang, Jialin Huang, Xiangli Ma, Juan Du, Yufen Bi, Peiwen Guo, Hao Lu, Lei Wang

**Affiliations:** ^1^ College of Landscape and Horticulture, Yunnan Agricultural University, Kunming, China; ^2^ School of Chemical Biology and Environment, Yuxi Normal University, Yuxi, China; ^3^ College of Animal Science and Technology, Yunnan Agricultural University, Kunming, China; ^4^ School of Mathematical Sciences, Xiamen University, Xiamen, China; ^5^ Scientific Research Department, Kunming Novo Medical Laboratory Co., Ltd., Kunming, China

**Keywords:** *Thalia dealbata*, near-complete genome assembly, PacBio HiFi, genome annotation, wetland plant

## Abstract

This study presents a chromosome-level, near-complete genome assembly of *Thalia dealbata* (Marantaceae), a typical emergent wetland plant with high ornamental and environmental value. Based on 36.99 Gb PacBio HiFi reads and 39.44 Gb Hi-C reads, we obtained a 255.05 Mb assembly, of which 251.92 Mb (98.77%) were anchored into eight pseudo-chromosomes. Five pseudo-chromosomes were completely assembled, and the other three had one to two gaps. The final assembly had a high contig N50 value (29.80 Mb) and benchmarking universal single-copy orthologs (BUSCO) recovery score (97.52%). The *T. dealbata* genome had 100.35 Mb repeat sequences, 24,780 protein-coding genes, and 13,679 non-coding RNAs. Phylogenetic analysis revealed that *T. dealbata* was closest to *Zingiber officinale*, whose divergence time was approximately 55.41 million years ago. In addition, 48 and 52 significantly expanded and contracted gene families were identified within the *T. dealbata* genome. Moreover, 309 gene families were specific to *T. dealbata*, and 1,017 genes were positively selected. The *T. dealbata* genome reported in this study provides a valuable genomic resource for further research on wetland plant adaptation and the genome evolution dynamics. This genome is also beneficial for the comparative genomics of Zingiberales species and flowering plants.

## 1 Introduction

Wetlands, also known as the “kidneys of the earth”, are of great ecological importance because they have played important roles in biodiversity conservation, carbon management, flood reduction, and water purification (Zedler and Kercher, 2005). Although wetlands cover less than 9% of the land area, they are vital habitats to many aquatic plants and animals (Gray et al., 2013). As key components of wetland ecosystems, wetland plants function as primary producers, habitats for other taxonomic groups, and nutrient removers (Cronk and Fennessy, 2016). Almost all wetland plants are angiosperms, with a few ferns and gymnosperms. These plants are categorized into emergent, submergent, floating-leaved, and floating plants based on their growth types and morphologies (Cronk and Fennessy, 2016). Although wetland plants have developed adaptation strategies to survive periodic soil saturation and the accompanying changes in soil chemistry (Pezeshki, 2001), the underlying genetic mechanisms in survival strategies are rarely studied. With the rapid development of sequencing technologies, the characterization of more wetland plant genomes can provide deeper insights into the adaptive evolution and morphological characteristics of wetland plants.

*Thalia dealbata* Fraser (Marantaceae), commonly known as powdery alligator flag, is a typical emergent wetland plant native to swamps and ponds in the Southern United States of America and Mexico. It has high ornamental value, given its long-stalked canna-like foliage and violet-blue flowers. This plant is usually covered with a white and water-repellent powdery coating, which enhances its performance. *T. dealbata* is also widely used in man-made wetlands to improve water quality by breaking down or removing excess pollutants from eutrophic water (Wang et al., 2020). A recent study has presented the complete chloroplast genome of *T. dealbata* (Deng et al., 2021). However, the *T. dealbata* nuclear genome has not been sequenced or reported.

Therefore, this study aimed to reconstruct a reference genome sequence of *T. dealbata* for further genomic and genetic studies. We performed a chromosome-level assembly of the *T. dealbata*, the first sequenced genome in the Marantaceae family, by integrating PacBio high-fidelity (HiFi) sequencing and chromosome conformation capture (Hi-C) technology. Subsequently, we performed a comparative genomics analysis of *T. dealbata* and other publicly available Zingiberales species, including *Musa acuminata* Colla (D’hont et al., 2012), *M. balbisiana* Colla (Wang et al., 2019), *Zingiber officinale* Roscoe (Li et al., 2021), and *Ensete glaucum* (Roxb.) Cheesman (Wang Z. et al., 2022). The reference-level genome assembly in this study will accelerate evolutionary and morphological studies of wetland plants and further phylogenomic studies of Marantaceae and Zingiberales.

## 2 Materials and methods

### 2.1 Sample collection and sequencing

Young and healthy *T. dealbata* leaves were collected from a mature *T. dealbata* plant growing on the lakeside of Dongan Lake in Chengdu, Sichuan Province, Southwest China. The leaves were immediately frozen in liquid nitrogen and stored at -80°C, awaiting further analysis.

High-quality total genomic DNA was extracted from the *T. dealbata* leaves using the CTAB method (Doyle and Doyle, 1987). For genome survey analysis, a paired-end library with an insert size of approximately 400 bp was constructed and sequenced on a NovaSeq 6000 platform. For the *de novo* assembly of the genome, SMRTbell libraries were prepared using the PacBio 15-kb protocol (Pacific Biosciences, CA, USA) and sequenced using the circular consensus sequencing (CCS) mode on the PacBio Sequel II sequencer. Finally, a Hi-C library was constructed and sequenced for the Hi-C scaffolding analysis on a NovaSeq 6000 platform.

In addition, the *T. dealbata* total RNA was extracted from the fresh stem, leaf, and flower tissues. Next, the RNA sequencing (RNA-seq) libraries were constructed and sequenced on an Illumina NovaSeq 6000 platform. The obtained raw RNA-seq reads were filtered using Trimmomatic (version 0.36) with default parameters (Bolger et al., 2014) for downstream genome annotation and quality assessment.

### 2.2 Genome survey

A *k*-mer (*k* = 19) analysis of Illumina short reads was performed using the Jellyfish (version 2.2.9, parameters, -k 19, -C) (Marçais and Kingsford, 2011). The low-frequency *k*-mers (frequency < 4) were removed, and the genome size was calculated by dividing the total *k*-mer number by the homozygous peak depth in the *k*-mer distribution curve. In addition, a polyploidy peak around the homozygous peak was examined to determine the ploidy level of the *T. dealbata* genome.

### 2.3 Genome assembly and quality assessment

HiFi long reads were pre-processed by CCS (version 4.2.0, parameters, min-passes = 3, min-length = 10, and min-rq = 0.99; http://ccs.how). Next, the filtered HiFi reads were assembled into contigs using hifiasm (version 0.14, default parameters) (Cheng et al., 2021) and mapped using Minimap (version 2.24, default parameters) (Li, 2018). Subsequently, the low-quality contigs with read depth < 10 or GC content > 50% were removed based on the GC-depth distribution. For Hi-C scaffolding analysis, Hi-C reads were mapped using Juicebox (version 1.8.8) with default parameters (Durand et al., 2016). Uniquely mapped Hi-C reads were then used to anchor contigs into chromosomes using 3D-DNA software (Dudchenko et al., 2017). Finally, scaffolding errors were checked and corrected according to the Hi-C contact heat maps generated with Juicebox.

The final assembly quality was evaluated by re-mapping the Illumina reads against the assembly using BWA (version 0.7.17) with default parameters (Li and Durbin, 2009). Subsequently, the benchmarking universal single-copy orthologs (BUSCO) completeness score was calculated by mapping 1,614 conserved genes from Embryophyta odb10 against the assembly using BUSCO (version 3.0.2) with default parameters (Simão et al., 2015). We searched the plant telomeres that are listed in the telomerase database (Podlevsky et al., 2007) against the final assembly using an in-house perl script. In addition, we identified centromeres within the *T. dealbata* genome using quarTeT (http://www.atcgn.com:8080/quarTeT/home.html) with the similar procedures which were described in Yue et al. (2023).

### 2.4 Identification of repeats

The repetitive elements in the *T. dealbata* genome were annotated using RepeatMasker (version v4.07) (Tarailo-Graovac and Chen, 2009) and RepeatModeler (version v1.0.11) (Price et al., 2005). First, a repeat library was *de novo* predicted based on the final assembly using RepeatModeler with default parameters. Next, a known repeat library of green plants was extracted using the “queryRepeatDatabase.pl” script from RepeatModeler. Finally, the two repeat libraries were combined into one comprehensive library, followed by a genome-wide homology-based identification of repeats using RepeatMasker. In addtion, we annotated intact long terminal repeat (LTR) retrotransposons (LTR-RTs) by integrating the predictions from LTR_Finder (version 1.06) (Xu and Wang, 2007) and LTRharvest (version 1.5.10) (Ellinghaus et al., 2008) using LTR_retriever (Ou and Jiang, 2018) with default parameters.

### 2.5 Annotation of protein-coding genes

Protein-coding genes were annotated as outlined by Wang et al. (2021). First, *E. glaucum*, *M. acuminata*, *M. balbisiana*, *Z. officinale*, and *Oryza sativa* L. protein sequences (Jain et al., 2019) were aligned with the *T. dealbata* genome using TBLASTN (version 2.2.31+, parameters, E-value < 1e-5) (Camacho et al., 2009), and a homology-based prediction was performed using GeneWise (version 2.4.1) with default parameters (Birney et al., 2004). Second, the *de novo* and genome-guided RNA-seq assemblies were combined for transcriptome-based prediction using the program to assemble spliced alignment (PASA; version 2.3.3) with default parameters (Haas et al., 2003). Third, a *de novo* prediction of gene models was performed using AUGUSTUS (version 3.2.3) (Stanke et al., 2006) with high-confidence gene model-trained parameters (exon number > 2 and CDS length > 1200 bp) selected from the PASA results. Finally, all the predicted gene models were integrated into a final gene set using EvidenceModeler (version 1.1.1) with default parameters (Haas et al., 2008). The final protein-coding gene set was functionally annotated using the publicly available protein databases, including Swiss-Prot, TrEMBL (Bairoch and Apweiler, 2000), InterPro (Hunter et al., 2009), and KEGG (Moriya et al., 2007), as described by Wang M. et al., (2022). Gene ontology (GO) terms were then assigned based on InterPro entries.

### 2.6 Annotation of non-coding RNAs

Non-coding RNAs (ncRNAs), including ribosomal RNAs (rRNAs), transfer RNAs (tRNAs), microRNAs (miRNAs), and small nuclear RNAs (snRNAs) were annotated using the *de novo* and homology-based methods. The rRNAs were predicted by aligning the assembly against the *Arabidopsis thaliana* rRNA sequences using BLASTN (version 2.2.31+, parameters, E-value < 1e-5). The tRNAs were predicted using tRNAscan-SE (version 1.4) (Lowe and Eddy, 1997), while snRNAs and miRNAs were predicted using Infernal (version 1.1.3) with default parameters (Nawrocki and Eddy, 2013).

### 2.7 Phylogenetic analysis and divergence time estimation

A phylogenetic analysis was performed based on the protein sequences of *T. dealbata* and four Zingiberales species, including *E. glaucum*, *M. acuminata*, *M. balbisiana*, and *Z. officinale* (haplotype A), with *O. sativa* as the outgroup species. The gene family clustering was performed using OrthoMCL (version 2.0.9) with default parameters (Li et al., 2003). Single copy genes (SCGs) in the six species were identified based on the clustering results. In addition, the SCGs protein sequences were aligned using MAFFT (version 7.313, parameters, LINSI) (Katoh et al., 2002). Finally, a maximum likelihood phylogenetic tree was reconstructed from the alignments of concatenated SCGs using RAxML (version 8.0.17, parameters, PROTGAMMAILGX, n = 500) (Stamatakis, 2014). The divergence time of *T. dealbata* was estimated using MCMCTREE in the PAML (version 4.9e, parameters, independent rates, F84 model) (Yang, 2007) based on the divergence between *O. sativa* and *E. glaucum* (103.2–117.1 million years ago, MYA) from the TimeTree database (Kumar et al., 2017) as the calibration point. Subsequently, the gene family expansions and contractions per species were detected using CAFE (version 3.1) with default parameters (De Bie et al., 2006).

### 2.8 Whole genome duplication (WGD) analysis

Recent WGD events within the *T. dealbata* genome were analyzed by comparing *T. dealbata* and *M. acuminata* protein sequences using MCScanX (version 1.1) with default parameters (Wang et al., 2012). Next, the synonymous substitution rate (*K*s) was calculated per collinear gene pair within and between the two species using “add_ka_and_ks_to_collinearity.pl”. Synteny blocks shared between *T. dealbata* and *M. acuminata* and in *T. dealbata* were visualized using TBtools (version 1.120) (Chen et al., 2020). Different gene duplication types were detected using DupGen_finder with default parameters (Qiao et al., 2019).

### 2.9 Selection analysis

Three species, including *T. dealbata*, *M. acuminata*, and *O. sativa* were selected for selection analysis. The coding sequences of SCGs among the three species were aligned using MAFFT and trimmed by Gblocks (version 0.91b) with default parameters (Castresana, 2000). Finally, selection analysis was performed based on the branch site model using Codeml in PAML (version 4.9e). The LRT p-value was calculated using Chi2 in PAML.

## 3 Results

### 3.1 Genome sequencing and assembly

A total of 21.30 Gb Illumina reads were generated for genome survey analysis (**Table S1**). The *T. dealbata* genome was estimated to be 256.15 Mb in size, with no evidence of polyploidy (**Figure S1**). In addition, 36.99 Gb (144.40× genome coverage) HiFi reads were generated for *de novo* genome assembly, which yielded 145 contigs with a total length of 260.54 Mb, which is very close to the estimated genome size. After removing the low-quality contigs with low read depth or high GC content (**Figure S2**), a Hi-C scaffolding analysis on 39.44 Gb Hi-C reads (153.97× genome coverage), yielded 251.92 Mb sequences that were anchored to eight pseudo-chromosomes (**Figure S3**).

**Figure 1:**
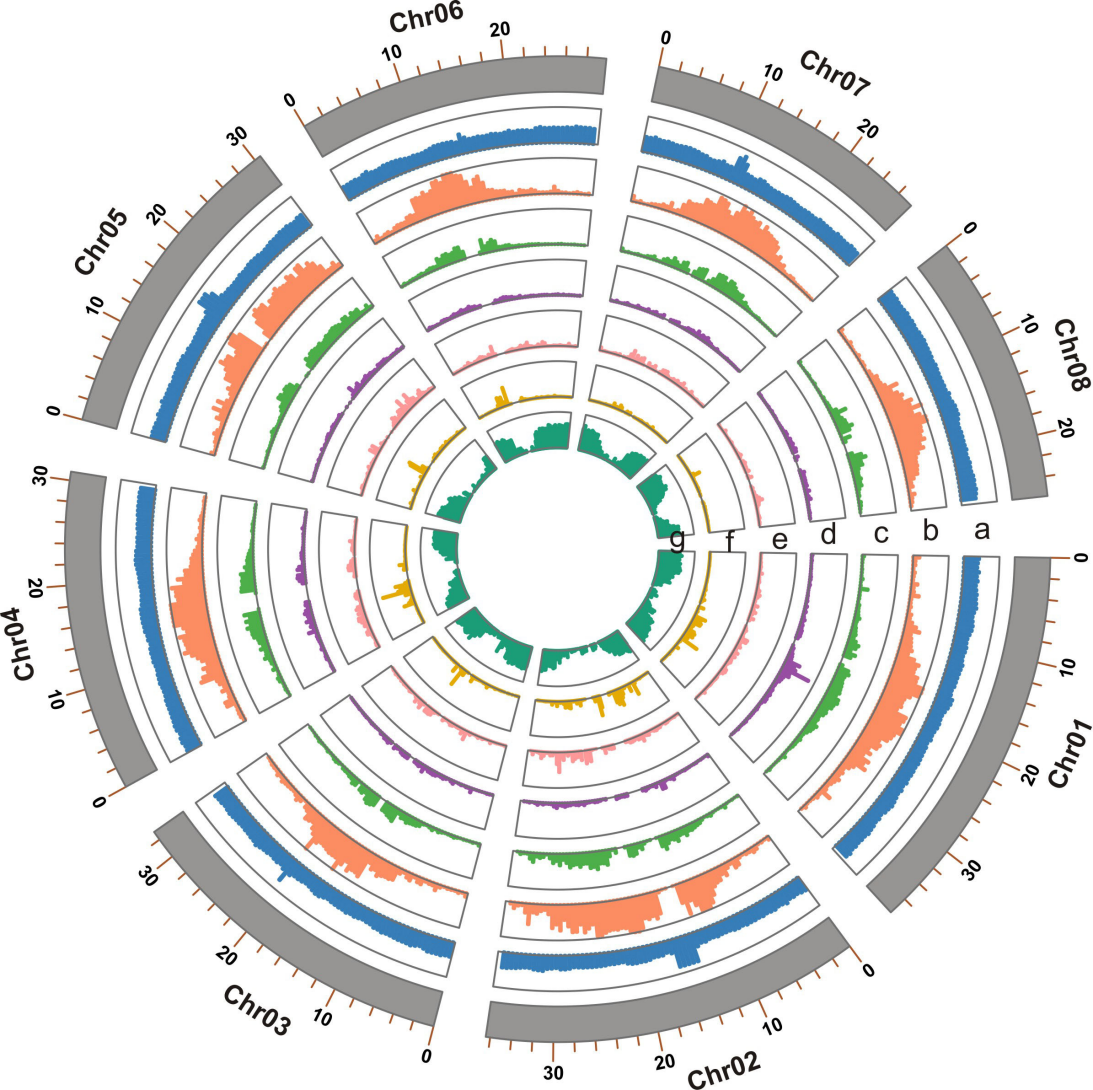
Genome features of the *T. dealbata* genome indicated in tracks from a to e: **(A)** GC content; **(B)** repeat content; **(C)** LTR density; **(D)** density of DNA transposons; **(E)** density of long interspersed nuclear elements; **(F)** density of short interspersed nuclear elements; and **(G)** gene density. All features are presented in non-overlapping windows of 500 kb.

The final genome assembly (**Figure 1**) was 255.05 Mb in length, with contig and super-scaffold N50 of 29.80 and 30.83 Mb, respectively (**Table 1**, **Table S2**). Five of the eight pseudo-chromosomes were completely assembled without any gap; two had one gap, while one pseudo-chromosome had two gaps (**Table S3**). Approximately 99.78% of the Illumina reads were mapped back to the *T. dealbata* genome, with a 10-fold minimum genome coverage of 99.87% (**Figure S4**). The genome assembly had an overall BUSCO score of 97.52% (**Table S4**). Approximately 96.34% of RNA-seq reads could be successfully aligned to the genome (**Table S5**). In addition, we found that all pseudo-chromosomes of the *T. dealbata* genome contained (TTAGGG)n telomeres at both ends (**Table S6**) and centromeres in the central region (**Table S7**). Overall, these data implied the *T. dealbata* genome was of high quality and completeness.

**Table 1:** Summary statistics for the *T. dealbata* assembly.

Genomic feature	Value
Estimated genome size (Mb)	256.15
Length of genome assembly (Mb)	255.05
Number of scaffolds	78
Longest scaffold (Mb)	37.82
Scaffold N50 (Mb)	30.83
Number of contigs	84
Longest contig (Mb)	37.82
Contig N50 (Mb)	29.80
Number of pseudo-chromosomes	8
Sequences anchored to pseudo-chromosomes (%)	98.77
Number of gaps	4
Numbers of gene models	24,780
Mean transcript length (bp)	3,352.15
Mean coding sequence length (bp)	1,289.94
Total size of repeat sequences (Mb)	100.35

### 3.2 Genome features

A total of 100.35 Mb of repeat sequences representing 39.34% of the *T. dealbata* genome were predicted from the high-quality assembly (**Table S8**). The repeat content of *T. dealbata* was lower than that of other sequenced Zingiberales species (**Figure S5**). The LTR-RTs were the most abundant repeat class, with 31.54 Mb (12.36%) of the genome and a *Gypsy* to *Copia* ratio of 4.4:1. Within the repeat-masked genome, 24,780 high confidence protein-coding genes covering 92.50% of the complete BUSCO genes were predicted (**Table S4**).

In addition, 24,020 (96.93%) gene models were assigned to known functions using at least one of the protein databases, with 15,611 (63.00%) assigned to GO terms (**Table S9**). At the same time, 13,679 ncRNAs with a total length of 2.09 Mb, including 5,358 rRNAs, 7,647 tRNAs, 152 miRNAs, and 522 snRNAs, were identified (**Table S10**).

### 3.3 Genome evolution

**Figure 2:**
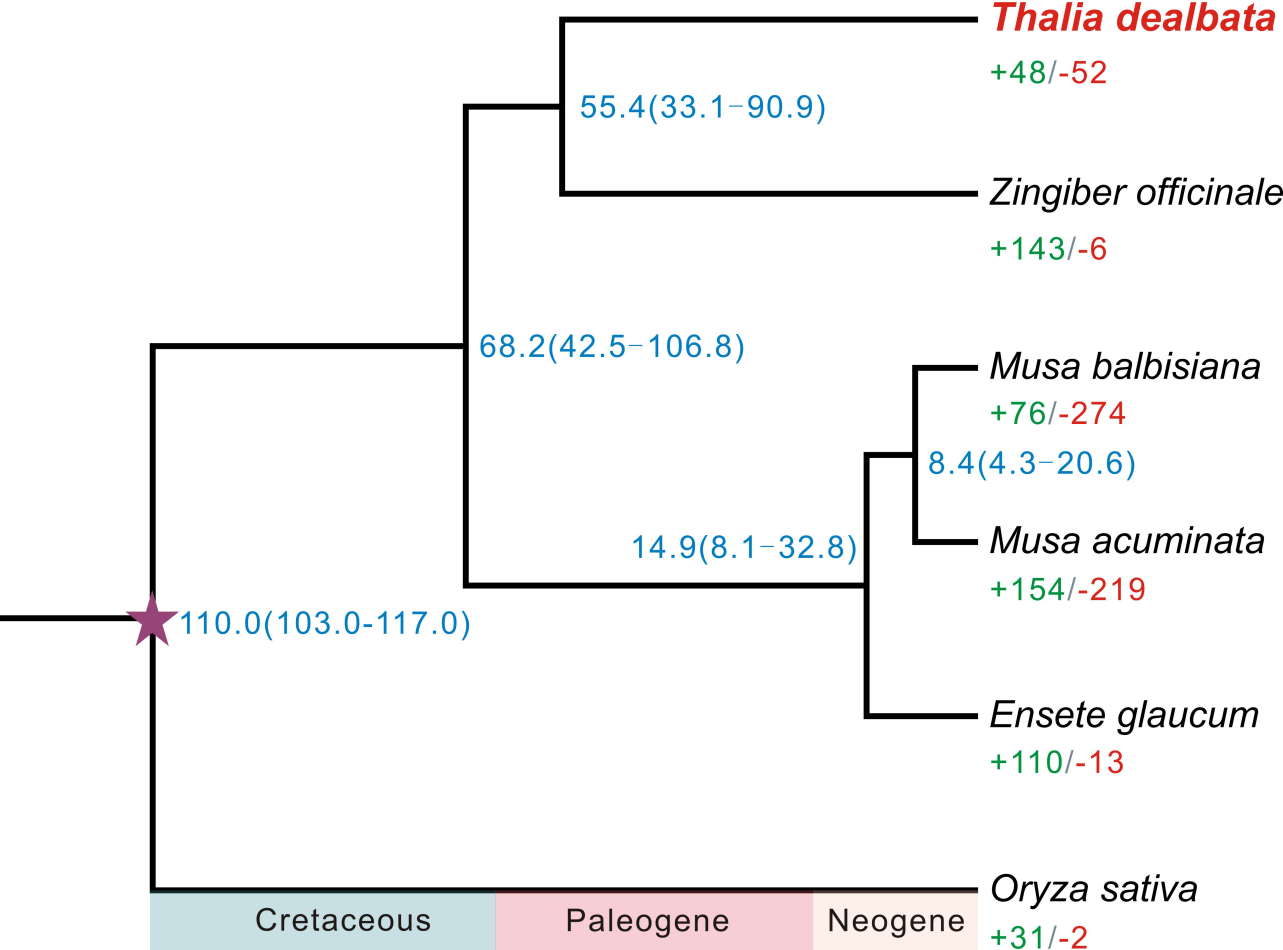
Phylogenetic tree of six plant species and evolution of gene families. The fully supported species tree was reconstructed based on 2,289 SCGs across the six species. Blue number beside each node shows the estimated divergence time of each node with 95% confidence intervals. The purple pentagram represent the calibration point. Green and red numbers indicate the numbers of significantly (*P* < 0.01) expanded and contracted gene families per species.

A total of 21,717 *T. dealbata* genes were classified into 13,613 families, 2,289 (16.81%) of which were located in the single-copy orthogroups across the six plant species (**Table S10**). A phylogenetic tree reconstructed based on the SCGs revealed that *T. dealbata* had the closest genetic relationship with *Z. officinale* (**Figure 2**). The divergence between *T. dealbata* and *Z. officinale* was estimated to be around 55.41 MYA. In addition, 48 and 52 gene families were significantly (*P* < 0.01) expanded and contracted in the *T. dealbata* genome, respectively, and 309 gene families containing 1,161 genes were specific to *T. dealbata* (**Table S11**). The 439 genes within the significantly expanded gene families were highly enriched in GO terms related to “glutathione metabolic process”, “response to wounding”, “photosynthesis, light reaction”, and “defense response” (**Figure S6**), which possibly contributes to *T. dealbata* adaption to wetland environments. In addition, the *T. dealbata* specific genes were functionally enriched in “intracellular transport”, “response to hormone”, “cell wall modification”, and “glycerolipid biosynthetic process” (**Figure S7**).

**Figure 3:**
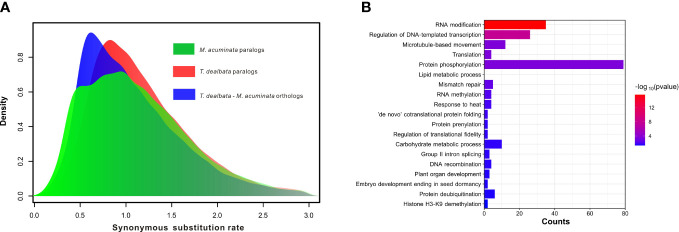
WGD event and positive selection in *T. dealbata.* **(A)** Results of recent WGD analysis based on the paralog and ortholog gene pairs within and between *T. dealbata* and *M. acuminata.* **(B)** GO enrichment of the 1,017 positively selected in *T. dealbata.*.

Furthermore, the WGD analysis revealed no recent WGD events in the *T. dealbata* genome (**Figure 3A**), although most (14,734; 59.46%) of the *T. dealbata* genes were classified as the WGD-derived genes (**Table S12**). However, the distribution of synonymous substitution rate (*K*s) showed that *T. dealbata* and *M. acuminata* shared an WGD event in their common ancestor. This ancient WGD event was also supported by the 2:2 relationship of the synteny blocks between *T. dealbata* and *M. acuminata* (**Figure S8**) and the 1:1 relationship of the synteny blocks within *T. dealbata* (**Figure S9**).

We detected a total of 764 intact LTR-RTs in the *T. dealbata* genome, all of which were inserted after the split of *T. dealbata* from *M. acuminata* (**Figure S10**). The *T. dealbata* genome contained significantly more intact LTR-RTs than the *M. acuminata* (764 vs 278), although *T. dealbata* had a smaller genome size than *M. acuminata* (255 Mb vs 473 Mb).

Finally, 3,863 SCGs were identified among *T. dealbata*, *M. acuminata*, and *O. sativa*, of which 1,017 genes were positively selected in *T. dealbata*. These positively selected genes were mainly related to “RNA modification”, “translation”, “lipid metabolic process”, “RNA methylation”, and “plant organ development” (**Figure 3B**).

## 4 Discussion and conclusion

In this study, we performed deep sequencing and chromosome-level genome assembly of *T. dealbata*, an emergent wetland plant belonging to Marantaceae from the order Zingiberales. Based on high coverage PacBio and Hi-C reads, we assembled a near-complete genome assembly of *T. dealbata*, the first reported assembly in Marantaceae. This *T. dealbata* assembly has considerably high completeness and continuity, with most of the pseudo-chromosomes were completely assembled. The high quality of this genome indicated the advantages of PacBio HiFi sequencing in constructing highly continuous genome assemblies with long and accurate reads (Nurk et al., 2020; Wang M. et al., 2022). However, there are still one to two gaps in three pseudo-chromosomes, which might be caused by the species-specific complex repetitive regions in *T. dealbata* genome. Further integration of ultra-long reads and other high-throughput sequencing data will make it possible to generate a telomere-to-telomere (T2T) genome for *T. dealbata*.

Using Hi-C scaffolding analysis, we anchored the genome sequences of *T. dealbata* into eight pseudo-chromosomes, showing the different chromosome number from a previous study (2n = 2x = 12; Suessenguth, 1921). The 3D-DNA software does not require *a priori* chromosome number as input, and the Hi-C contact map shows a clear pattern of eight chromosome interaction (**Figure S3**). Thus, we speculated that there was some mistake in the relatively old research of Suessenguth (1921). Future karyotype analysis of *T. dealbata* can verify the validity of our speculation.

The high-quality *T. dealbata* genome has lead to accurate structural annotation of pretein-coding genes and ncRNAs, enabling us to gain further insights into the evolutionary history of *T. dealbata* and related species. We found that *T. dealbata* had the closest genetic relationship with *Z. officinale*, which was consistent with the close relationship between Marantaceae and Zingiberaceae that was revealed by two previous studies (Sass et al., 2016; Carlsen et al., 2018). These two species shared an ancient WGD event with *M. acuminata* in their common ancestor, which was follwed by diploidization events that involved substantial genome reshuffling and gene losses. The early divergence among these species provided a long enough period to allow sufficient divergence in genomic characteristics and adaptation strategies in *T. dealbata* and *Z. officinale.* The identified expanded, contracted, and unique gene families together with a number of positively selected genes in *T. dealbata* genome are possibly responsible to the adaptation of *T. dealbata* to wetland environment.

Overall, the high-quality *T. dealbata* genome assembly presented in this study will provide a valuable genomic resource for the study of plant adaptation to wetland environments and the evolutionary analysis of Marantaceae and Zingiberales. We look forward more genetic and genomic analysis and functional studies of this interesting wetland plant in the future.

## Data availability statement

The datasets presented in this study can be found in online repositories. The names of the repository/repositories and accession number(s) can be found in the article/**Supplementary Material**.

## Author contributions

MT, JH, LW, and HL designed and supervised the study. MT and JD collected the samples and extracted the genomic DNA and RNA. MT, XM, and PG performed genomic data analysis. MT drafted the manuscript, and YB revised this manuscript. All authors contributed to the article and approved the submitted version.
