# Supplementary material for: A near-complete genome assembly of Thalia dealbata Fraser (Marantaceae)
Source: Front Plant Sci. 2023 Jun 13;14:1183361. doi: 10.3389/fpls.2023.1183361 (PMC10298163; doi:10.3389/fpls.2023.1183361)
Supplement: Supplementary file 1 [file DataSheet_1.pdf]

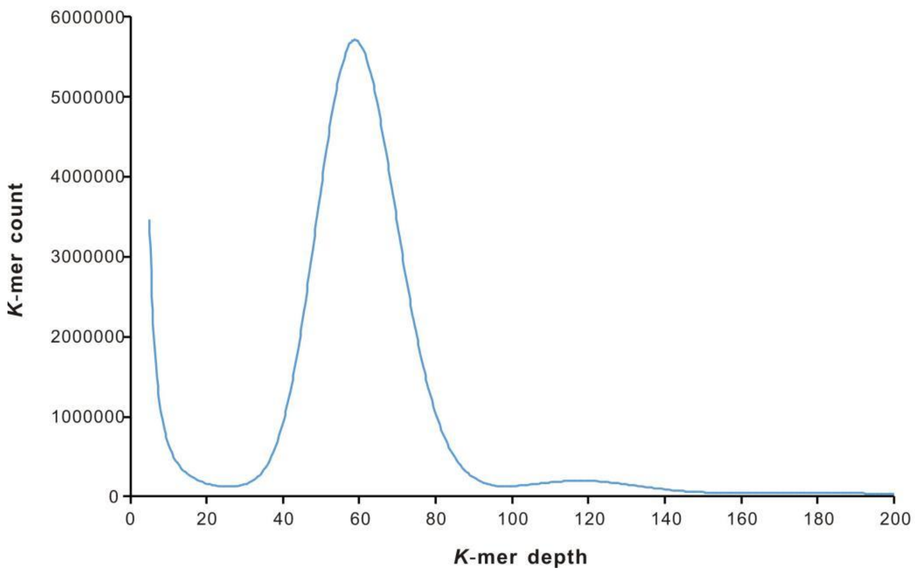

Figure S1. Histogram of 19-mer analysis for the *T. dealbata* genome.

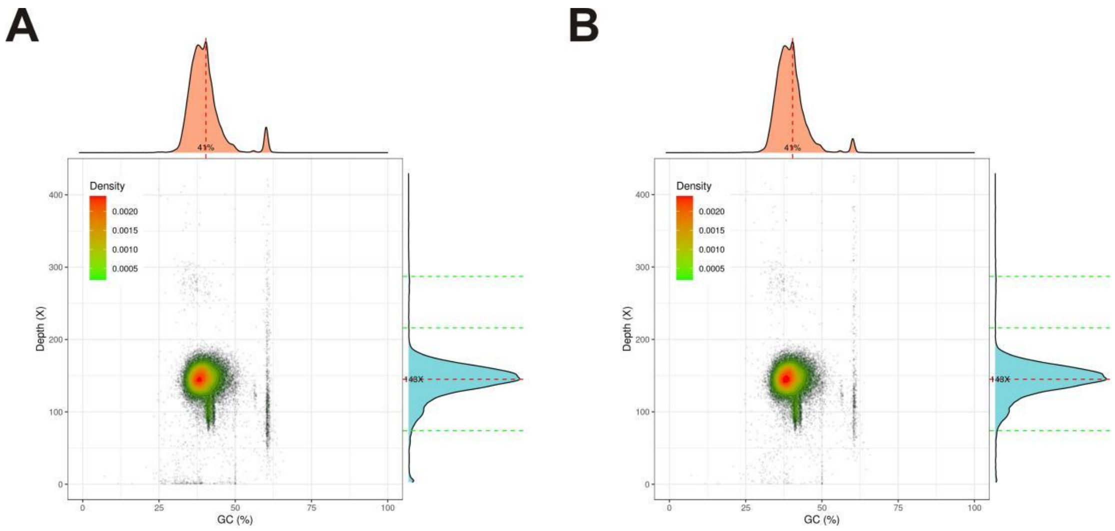

Figure S2. GC-depth distribution maps before (A) and after (B) removing low-quality contigs in the *T. dealbata* assembly.

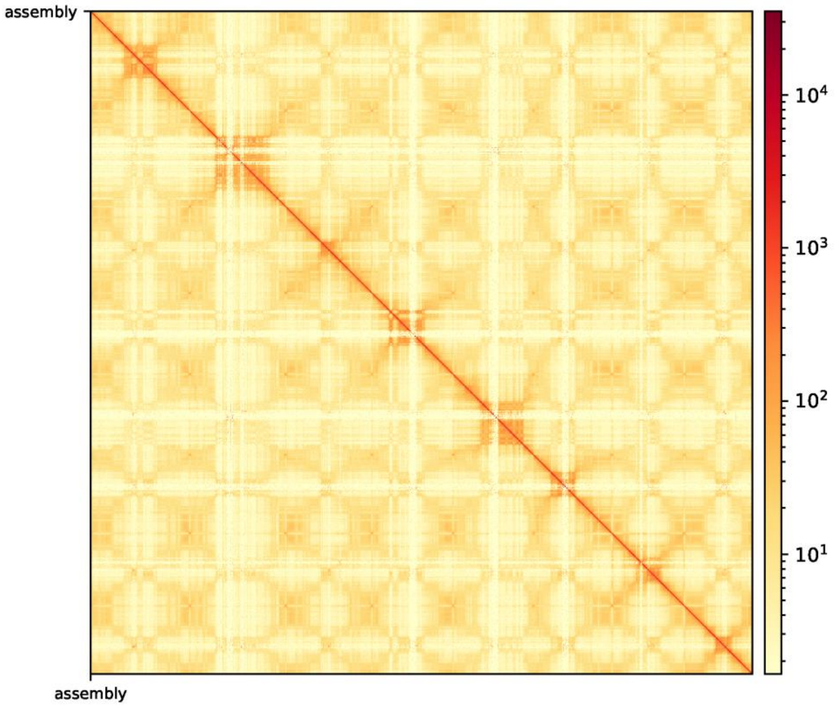

Figure S3. Heatmap showing the Hi-C interactions of all chromosomes of *T. dealbata*.

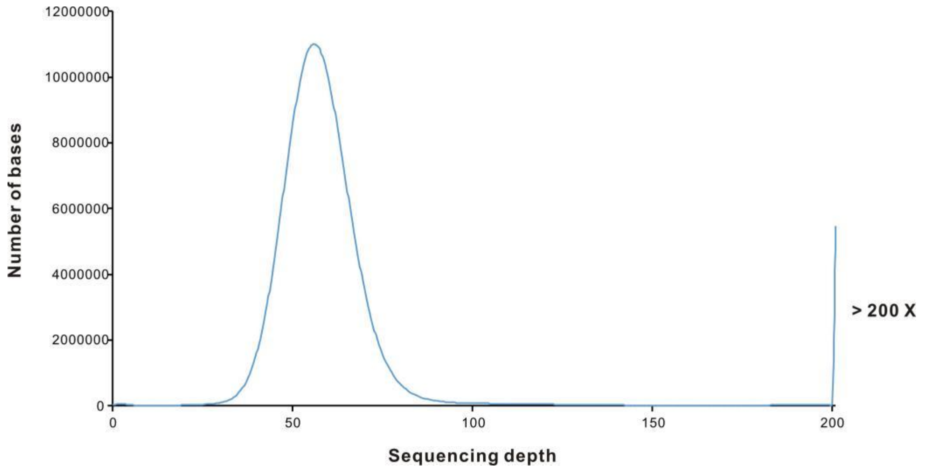

Figure S4. Read depth distributions obtained by mapping Illumina reads against the *T. dealbata* genome.

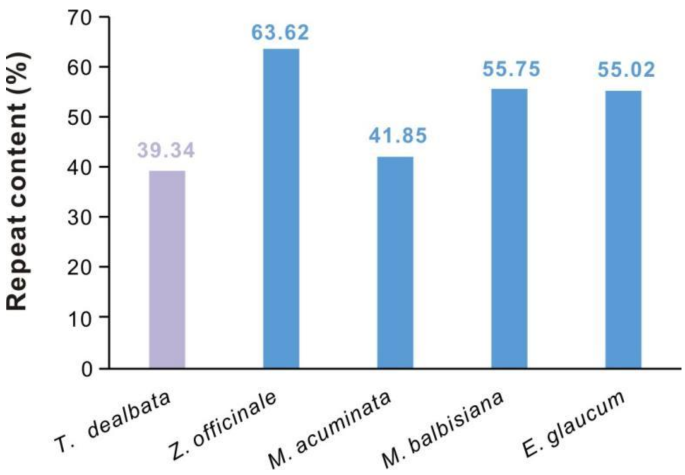

Figure S5. Repeat content of *T. dealbata* and four sequenced Zingiberales species.

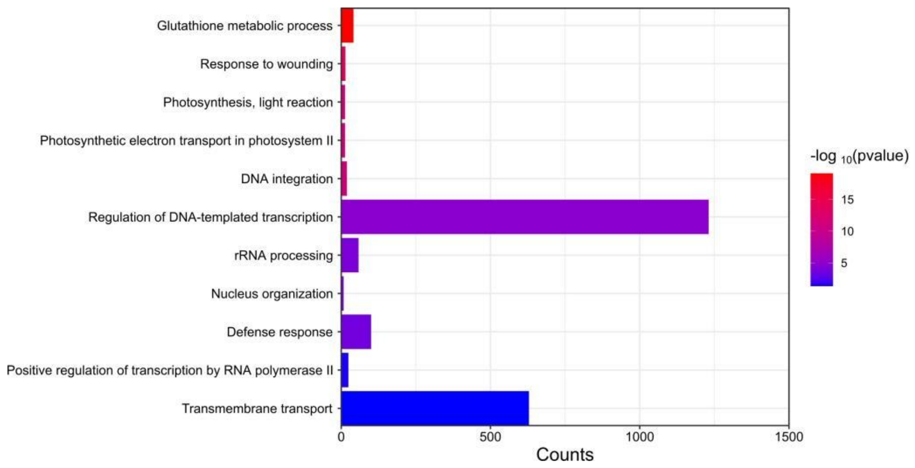

Figure S6. GO enrichment of significantly expanded gene families in *T. dealbata.*

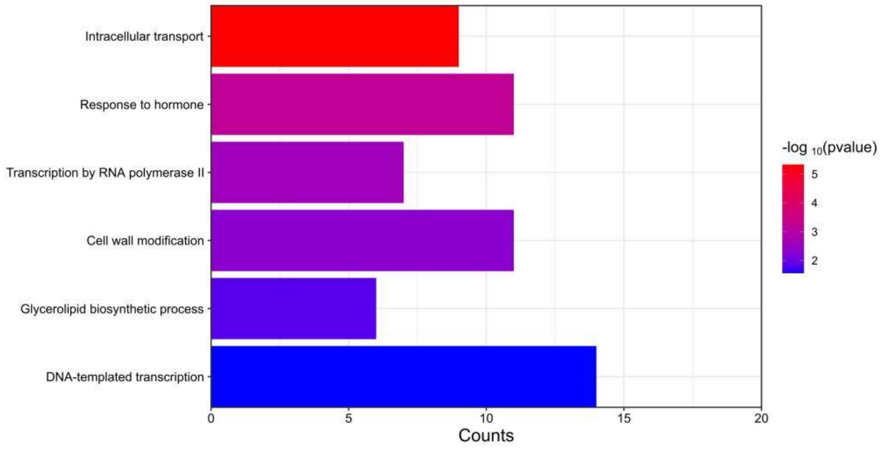

Figure S7. GO enrichment of unique gene families in *T. dealbata.*

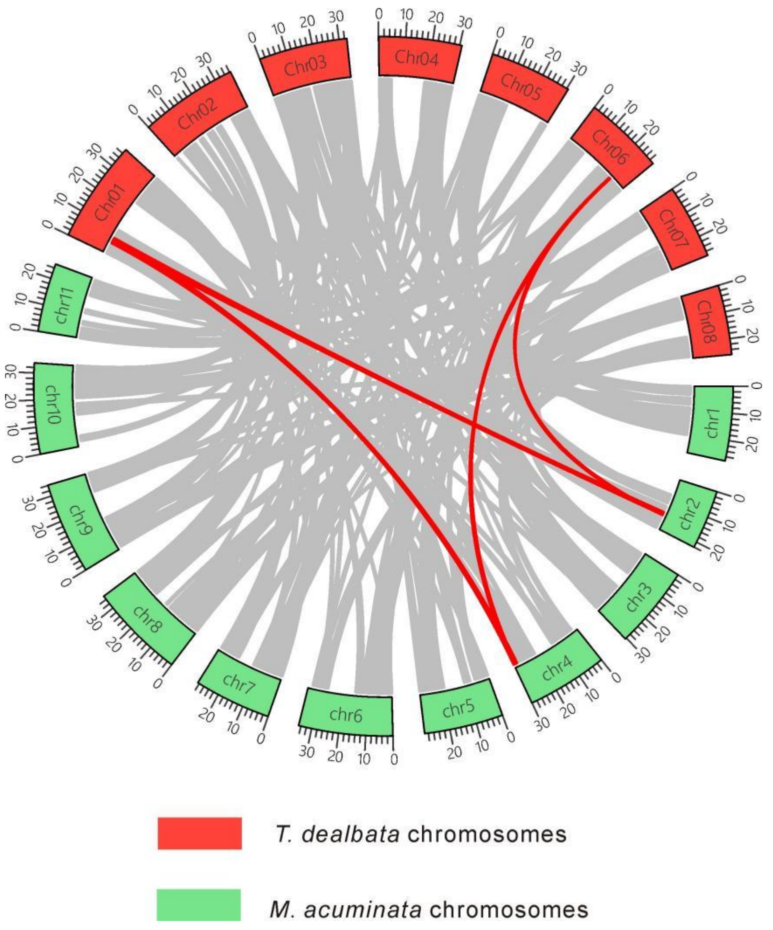

Figure S8. Synteny blocks between*T. dealbata* and *M. acuminata.* Typical blocks showing 2:2 relationship are marked in red.

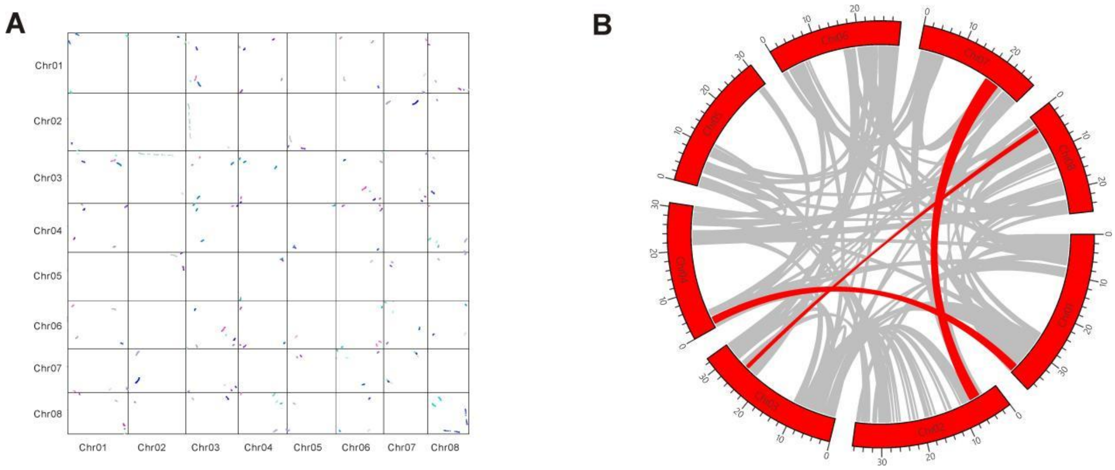

Figure S9. Dot plot (A) and synteny blocks (B) among chromosomes of *T. dealbata*. Typical blocks showing 1:1 relationship were marked in red.

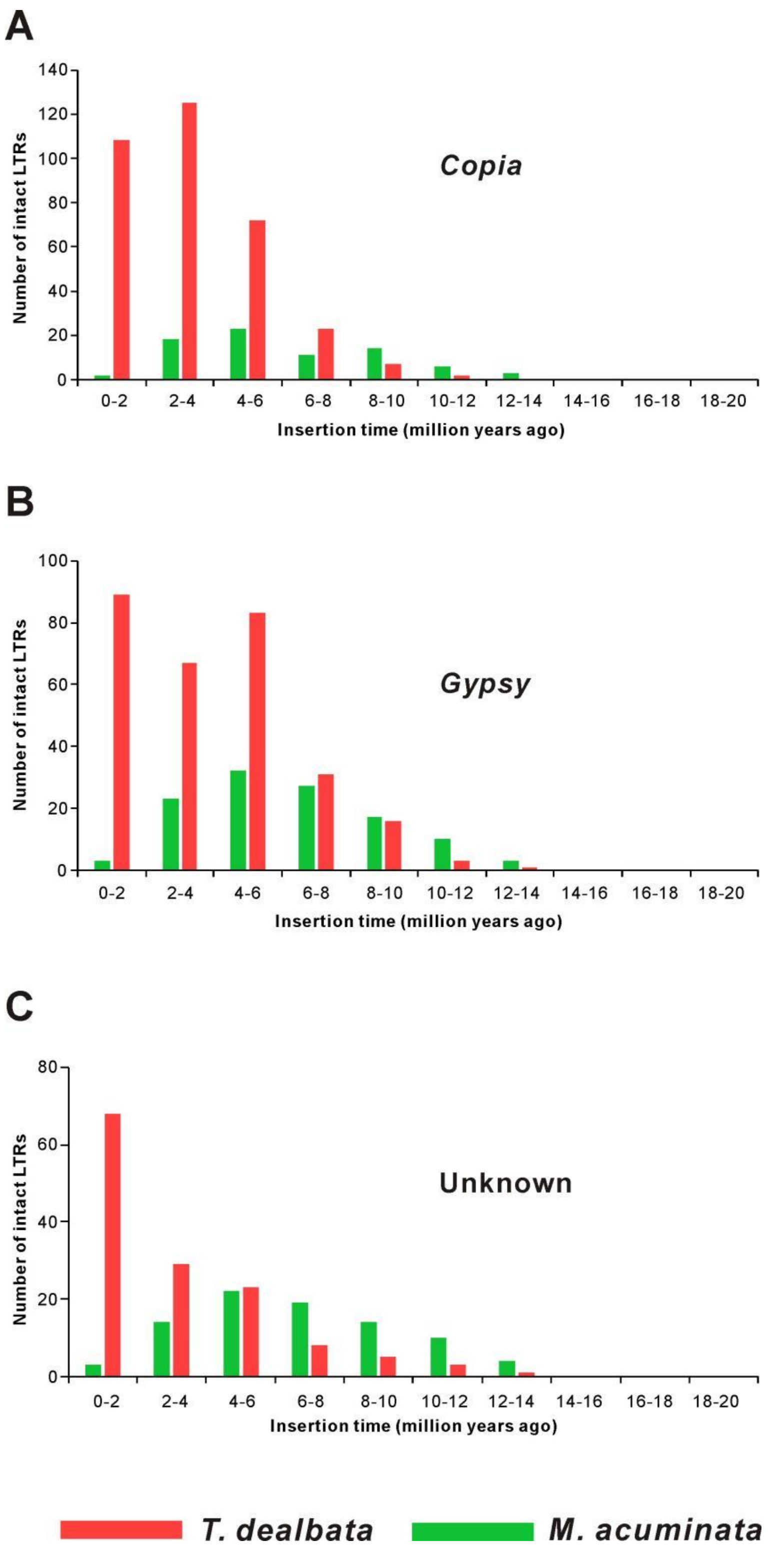

Figure S10. Insert time distribution of intact LTR-RTs in the genomes of *T. dealbata* and *M. acuminata.*

**Platform**	**Illumina**	**HiFi**	**Hi-C**
Read length (bp)	150	-	150
Number of reads	81,094,260*2	2,137,534	131,464,319*2
Number of bases (bp)	21,298,954,500	36,989,899,018	39,439,295,700
Sequence coverage (×)*	81.35	144.40	153.97

* The estimated genome size was ~256.15 Mb.

Table S1. Summary of Illumina, PacBio HiFi and Hi-C reads for the assembly of *T. dealbata* genome.

	Contig		Scaffold	
	**Size (bp)**	**Number**	**Size (bp)**	**Number**
N50	29,804,147	4	30,829,658	4
N60	25,874,500	5	30,634,904	5
N70	21,629,495	6	29,804,147	6
N80	15,989,488	8	27,648,310	7
N90	11,391,480	10	25,874,500	8
Longest	37,817,525	-	37,817,525	-
Number > 1 Mb	-	11	-	8
Number > 5 Mb	-	11	-	8
**Total**	**255,046,420**	**84**	**255,049,420**	**78**

Table S2. Summary of the final *T. dealbata* genome.

Chromosome ID	Length (bp)	Contig number	Gene number
Chr01	37,817,525	1	3,835
Chr02	36,425,697	1	2,614
Chr03	32,886,241	2	3,992
Chr04	30,829,658	1	2,939
Chr05	30,634,904	2	2,229
Chr06	29,804,147	1	3,441
Chr07	27,648,310	3	2,819
Chr08	25,874,500	1	2,872
**Total**	**251,920,982**	**12**	**24,741**

Table S3. Summary of the eight chromosomes of the *T. dealbata* genome.

	Assembly		Annotation	
	**Count**	**Ratio (%)**	**Count**	**Ratio (%)**
Complete BUSCOs	1,574	97.52	1,493	92.50
Complete and single-copy BUSCOs	1,532	94.92	1,443	89.41
Complete and duplicated BUSCOs	42	2.60	50	3.09
Fragmented BUSCOs	9	0.56	38	2.35
Missing BUSCOs	31	1.92	83	5.14
Total BUSCOs	1,614	100.00	1,614	100.00

Table S4. BUSCO recovery scores of the *T. dealbata* genome assembly and annotation.

**Tissue**	**Number of reads**	**Total size (bp)**	**Mapping rate (%)**	**Accession number**
Leaf	43,081,002	6,365,689,200	95.73	SRR23717304
Stem	40,383,180	5,977,837,106	97.01	SRR23717303
Flower	53,894,038	7,960,821,535	96.33	SRR23717302
**Total**	137,358,220	20,304,347,841	96.34	-

Table S5. Summary of RNA-seq data used in this study.

Chromosomes	Type	Start position	Length (bp)
Chr01	TTAGGG	330,194	12
Chr01	TTAGGG	37,316,003	12
Chr02	TTAGGG	1,718,990	12
Chr02	CCCTAA	36,146,519	12
Chr03	TTAGGG	353,429	12
Chr03	CCCTAA	31,333,942	12
Chr04	TTAGGG	228,324	12
Chr04	CCCTAA	30,561,781	18
Chr05	CCCTAA	1,048,645	12
Chr05	CCCTAA	30,409,580	12
Chr06	TTAGGG	1,036,489	24
Chr06	CCCTAA	29,476,268	12
Chr07	TTAGGG	1,302,342	24
Chr07	TTAGGG	27,056,277	12
Chr08	CCCTAA	1,461,237	12
Chr08	CCCTAA	25,515,820	12

Table S6. Candidate telomere sequences in the *T. dealbata* genome assembly.

Chromosomes	Start position	End position	Length (bp)	TR length (bp)
Chr01	15,057,795	17,810,270	2,752,476	1,680,782
Chr02	18,286,156	21,896,474	3,610,319	2,446,765
Chr03	15,623,622	17,069,740	1,446,119	1,078,521
Chr04	12,861,089	13,602,284	741,196	498,042
Chr05	13,532,755	17,435,525	3,902,771	2,038,645
Chr06	8,586,473	13,686,840	5,100,368	2,735,803
Chr07	13,326,199	16,765,042	3,438,844	1,278,434
Chr08	14,143,270	16,333,165	2,189,896	2,113,915

Table S7. Candidate centromere sequences in the *T. dealbata* genome assembly.

Type	Total length (bp)	Percent of genome (%)
DNA	5,319,378	2.09
LINE	2,979,798	1.17
SINE	4,080,496	1.60
LTR	31,535,323	12.36
Satellite	1,855,077	0.73
Simple repeat	8,479,454	3.32
Low complexity	1,942	0.00
Unknown	47,296,758	18.54
**Total**	**100,347,734**	**39.34**

Table S8. Summary of repeat sequences within the *T. dealbata* genome.

	Number of genes	Percent (%)
Total	24,780	-
Annotated	24,020	96.93
InterPro	23,926	96.55
KEGG	8,803	35.52
SwissProt	17,102	69.02
TrEMBL	22,607	91.23
GO	15,611	63.00
Unannotated	760	3.07

Table S9. Functional annotation of the protein-coding genes in the *T. dealbata* genome using publicly available protein databases.

**Type**	**Number**	**Average length (bp)**	**Total length (bp)**
miRNA	152	130.74	19,873
tRNA	7,647	75.71	578,987
rRNA	5,358	266.04	1,425,432
28S	1,413	124.38	175,745
18S	501	1,662.66	832,994
5.8S	461	150.67	69,457
5S	2,983	116.40	347,236
snRNA	522	131.31	68,546
CD-box	228	106.85	24,361
HACA-box	46	131.76	6,061
Splicing	248	153.73	38,124

Table S10. Summary of ncRNAs in the *T. dealbata* genome assembly.

	Genes number	Genes in families	Unclustered genes	Family number	Genes per family	Unique families	Unique families genes
Thalia dealbata	24,780	21,717	3,063	13,613	1.82	309	1,161
Musa acuminata	36,528	30,121	6,407	16,305	2.24	293	749
Musa balbisiana	33,021	28,168	4,853	15,931	2.07	244	796
Zingiber officinale	31,821	29,741	2,080	13,593	2.34	656	3,802
Ensete glaucum	36,836	30,091	6,745	16,306	2.26	216	583
Oryza sativa	35,594	27,628	7,966	14,157	2.51	1,686	7,742

Table S11. Summary of gene family clustering between *T. dealbata* and five other plant species.

	**Tandem**	**Transposed**	**Proximal**	**Dispersed**	**WGD**
Thalia dealbata	1,388	2,535	750	1,517	14,734
Musa acuminata	856	2,628	628	3,205	20,023

Table S12. Numbers of genes belonging to different duplication types in *T. dealbata* and *M. acuminata.*
